# Limited Neonatal Carbohydrate-Specific Antibody Repertoire Consecutive to Partial Prenatal Transfer of Maternal Antibodies

**DOI:** 10.3389/fimmu.2020.573629

**Published:** 2020-10-14

**Authors:** Katharina Kappler, Tanja Restin, Yi Lasanajak, David F. Smith, Dirk Bassler, Thierry Hennet

**Affiliations:** ^1^Institute of Physiology, University of Zurich, Zurich, Switzerland; ^2^Department of Neonatology, University Hospital Zurich, University of Zurich, Zurich, Switzerland; ^3^Emory Comprehensive Glycomics Core, Department of Biochemistry, Emory University School of Medicine, Atlanta, GA, United States

**Keywords:** immunoglobulin, glycosylation, glycan, microarray, microbiota, bacteroides

## Abstract

Despite the prominence of carbohydrate-specific antibodies in human sera, data on their emergence and antigen specificities are limited. Whereas maternal IgG are transferred prenatally to the fetal circulation, IgM present in cord blood originate from fetal B lymphocytes. Considering the limited exposure of the fetus to foreign antigens, we assessed the repertoire of carbohydrate-specific antibodies in human cord blood and matched maternal blood samples using glycan arrays. Carbohydrate-specific IgM was absent in cord blood, whereas low cord blood IgG reactivity to glycans was detectable. Comparing IgG reactivities of matched pairs, we observed a general lack of correlation in the antigen specificity of IgG from cord blood and maternal blood due to a selective exclusion of most carbohydrate-specific IgG from maternofetal transfer. Given the importance of intestinal bacteria in inducing carbohydrate-specific antibodies, we analyzed global antibody specificities toward commensal bacteria. Similar IgG reactivities to specific *Bacteroides* species were detected in matched cord and maternal blood samples, thus pointing to an efficient maternal transfer of anti-microbial IgG. Due to the observed selectivity in maternofetal IgG transfer, the lack of fetal antibodies to carbohydrate epitopes is only partially compensated by maternal IgG, thus resulting in a weak response to carbohydrate antigens in neonates.

## Introduction

Carbohydrate-specific antibodies represent a large fraction of circulating IgM and IgG. These antibodies mainly recognize alloantigens, such as ABO and Lewis blood groups (1, 2), and xenoantigens, including α-rhamnose, Forssman, and Galili antigens (3–5). Whereas allo-antigen specific carbohydrate-specific antibodies are relevant in transfusion medicine, carbohydrate-specific antibodies generally protect against infections by targeting carbohydrate antigens expressed by pathogens, such as bacterial capsular polysaccharides (6) and surface glycoproteins of parasites (7).

Carbohydrate-specific antibodies develop early in life (8, 9), mainly following the colonization of the gastrointestinal tract with microbes, which stimulate the infant's immune system (10). ABO-specific antibodies typically occur postnatally as the result of the exposure to bacterial glycans mimicking ABO antigens (11). The transfer of maternal IgG through the placenta provides an additional level of protection to the newborn. IgG is the only immunoglobulin class that can pass the placenta by binding to the neonatal Fc receptors (FcRn) expressed at the syncytiotrophoblast (12). At time of birth, IgG levels in cord blood reach adult levels and often even exceed maternal levels (13). Most cord blood IgG are considered to be of maternal origin (14). Maternally-transferred IgG have been mainly investigated in the context of vaccine protection toward *Bordetella pertussis* (15), *Staphylococcus* (16), *Haemophilus influenzae* (17), and *Streptococcus pneumoniae* (18). Differences in the efficiency of placental antibody transfer may relate to antigen properties and the IgG subclass involved (19). Antibodies to bacterial polysaccharides are mainly of IgG2 subclass, which is less efficiently transferred to the fetal circulation through FcRn (20). On the contrary, the cord blood levels of IgG to xenoantigens, such as α-rhamnose and the Forssman antigen, and to bacterial lipopolysaccharides from *Escherichia coli* O16, O6, and O111 have been shown to be at least as high as in matched maternal samples (21, 22).

In contrast to IgG, IgM levels in cord blood are low (23, 24), as they are solely derived from the developing immune system of the fetus (25). These IgM are classified as natural antibodies without antigen-driven maturation (26), although pre-natal immune stimulation by antigens transferred from the maternal circulation (27, 28) or by bacteria occurring *in utero* have to be considered. The presence of bacteria *in utero*, however, is controversial (29) as the fetal environment is largely considered to be sterile (30, 31). Isolated studies reported the presence of bacteria in meconium (32), amniotic fluid (33), cord blood (34), and placental tissue (33, 35). Accordingly, the occurrence of cord blood IgM targeting bacterial glycoconjugates and carbohydrates in general is unclear. IgM to some glycans have been described in cord blood (26), whereas other studies reported that carbohydrate-specific antibodies are absent during the first weeks of life (36).

To characterize the repertoires of maternally transferred and endogenous carbohydrate-specific antibodies at birth, we compared the reactivity of IgG and IgM toward carbohydrate antigens in matched maternal and cord blood samples. The analysis of antibody binding to an array of human oligosaccharides pointed to striking differences in the reactivity of maternal and neonatal antibodies toward carbohydrate antigens.

## Results

### Carbohydrate-Specific IgG and IgM in Cord Blood and Maternal Blood

**Figure 1:**
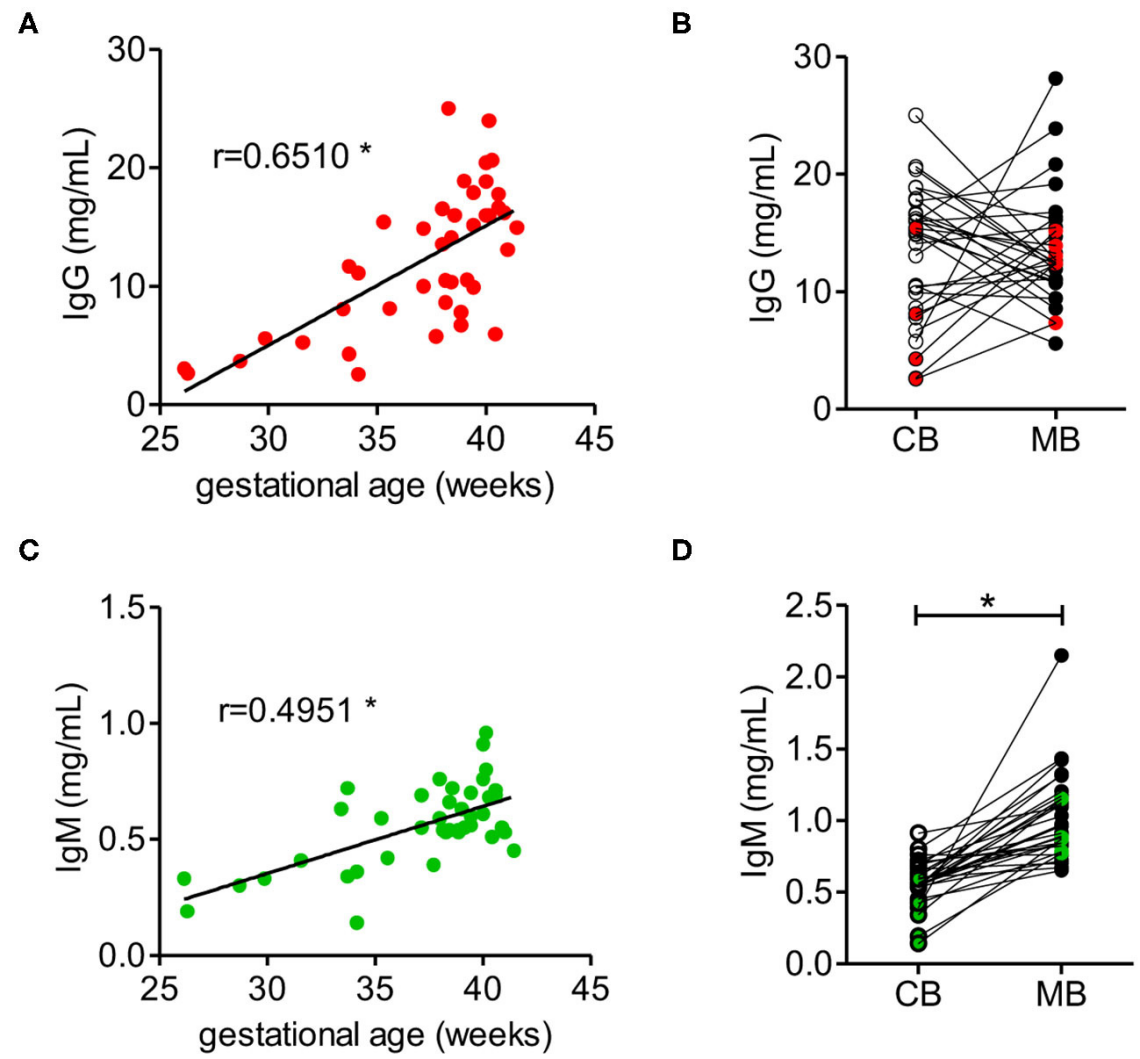
IgM and IgG concentrations in cord blood depend on the gestational age. **(A)** IgG concentration of cord blood samples dependent on the gestational age (*n* = 42). **(B)** IgG concentrations of cord blood (CB) and matched maternal blood (MB) (*n* = 31). Red dots indicate samples from pre-term neonates (*n* = 5). **(C)** IgM concentrations of cord blood dependent on the gestational age (*n* = 42). **(D)** IgM concentrations of cord blood (CB) and maternal blood (MB) (*n* = 31). Green dots indicate samples from pre-term neonates (*n* = 5). Results with *p*-value < 0.05 are marked with asterisks.

The concentrations of IgG and IgM in cord blood increased with gestational age, where the IgG concentration in cord blood from full-term neonates with a gestational age of at least 37 weeks reached between 10 and 20 mg/ml, which is within the range of IgG concentrations in maternal blood (Figure 1A, Supplementary Table 1). Direct comparison of matched cord and maternal blood samples showed no correlation in respect to IgG concentration. Notably, high IgG concentrations in maternal blood were mainly associated with lower concentration in matched cord blood samples (Figure 1B).

IgM concentration in cord blood also increased with gestational age (Figure 1C), although at term IgM concentration remained significantly lower than the concentration of about 1 mg/ml measured in maternal blood (Figure 1D, Supplementary Table 1). In contrast to IgG, the levels of IgM in neonatal cord blood were consistently lower than the serum IgM concentration in the matched maternal blood samples (Figure 1D).

To compare the antigenic repertoire of carbohydrate-specific antibodies in matched cord blood and maternal blood samples, we focused on neonates delivered at full term to maintain consistency in IgM and IgG levels. The sample population investigated comprised 26 pairs of matched cord blood and maternal blood samples. IgG concentrations in this group of samples averaged 14.6 ± 4.7 mg/ml for cord blood samples and 13.7 ± 5.0 mg/ml for maternal blood samples. IgM concentrations were 0.6 ± 0.1 mg/ml in cord blood samples and 1.0 ± 0.3 mg/ml in maternal blood samples.

Carbohydrate antigen specificity of IgG and IgM in matched cord blood and maternal blood samples was investigated using an array displaying 220 different human oligosaccharides (37). The oligosaccharide structures tested represented common carbohydrate epitopes found on human cells and occurring on bacterial glycoconjugates expressed by intestinal commensals (11, 38). Most oligosaccharides displayed on the array were characterized by their mass composition consisting of hexose (H), N-acetyl-hexosamine (N), fucose (F) and the sialic acid N-acetylneuraminic acid (S). Antibody binding to oligosaccharides was calculated as the mean fluorescence intensity of four replicates for each oligosaccharide.

**Figure 2:**
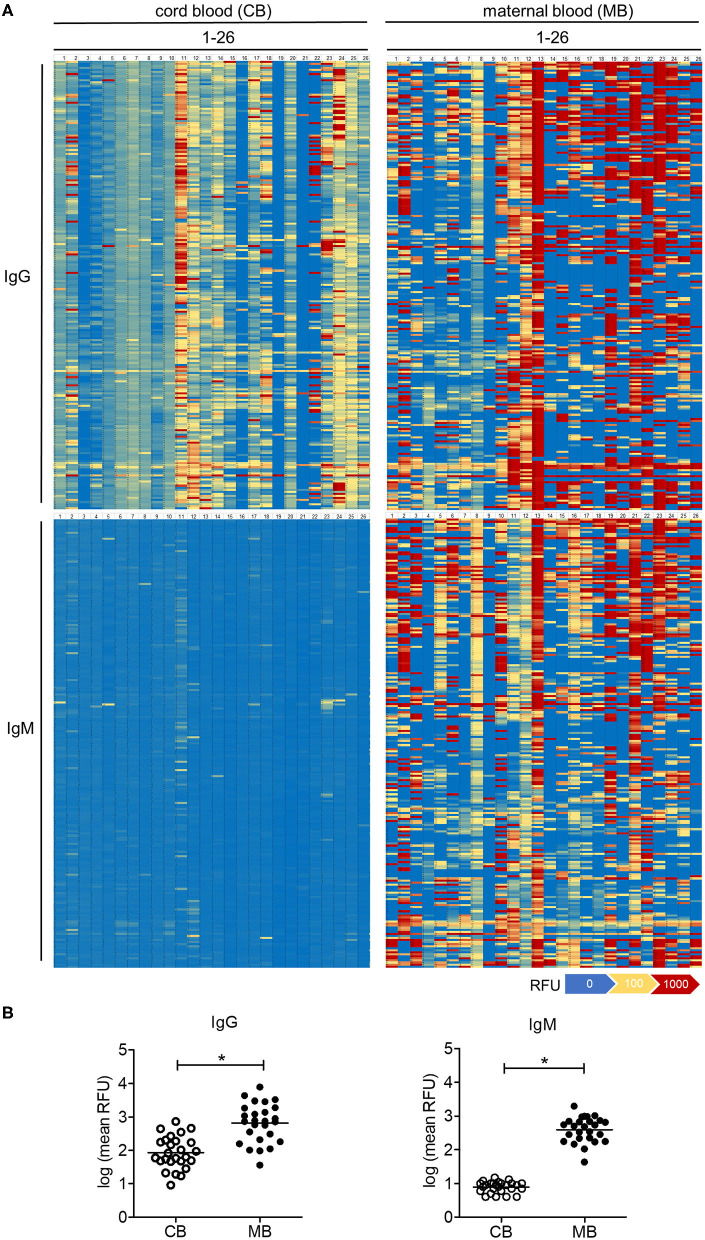
Carbohydrate-specific IgG is lower in cord blood than in matched maternal blood, whereas carbohydrate-specific IgM is absent in cord blood. **(A)** Heatmaps of the serum IgG and IgM reactivity to 220 different glycans measured by glycan array analysis, displayed for 26 matched pairs of cord blood (CB1-26) and maternal blood (MB1-26). **(B)** Quantification of cord blood (CB) and maternal blood (MB) IgG and IgM reactivity to glycans measured by arrays. Results with *p*-value < 0.05 are marked with asterisks.

The comparative analysis of 26 maternal blood samples revealed a large inter-individual variability in the range and intensity of oligosaccharide binding by IgG across samples (Figure 2A). Whereas isolated samples showed a strong reactivity toward oligosaccharides (samples MB13, MB21 on Figure 2A), others showed only a minor reactivity to few oligosaccharides (samples MB4, MB9 on Figure 2A). The difference in the average binding intensity to oligosaccharides exceeded a 100-fold between the least and the most reactive maternal blood samples (Figure 2B). The reactivity of IgG to oligosaccharides in cord blood samples was on average 10-fold lower than in the maternal blood samples, whereas a large inter-individual difference was also evident between the samples tested (Figure 2A).

Given that the placental transfer of antibodies is restricted to the IgG class, the amount and reactivity of IgM detected in cord blood samples reflect the endogenous production by the fetal immune system (25). In contrast to IgG, cord blood IgM largely did not react with the oligosaccharide antigens on the array (Figure 2A). Maternal blood IgM showed a strong reactivity to oligosaccharides in a similar way to the signals recorded for IgG (Figure 2A). Individual recognition patterns and signal intensities for IgG and IgM of the same serum strongly overlapped (Supplementary Figure 1). Correlation analysis confirmed that repertoires of carbohydrate-specific IgG and IgM correlate significantly for each maternal blood serum (Supplementary Table 1), clearly showing the lack of cord blood IgM reactivity toward oligosaccharides.

**Figure 3:**
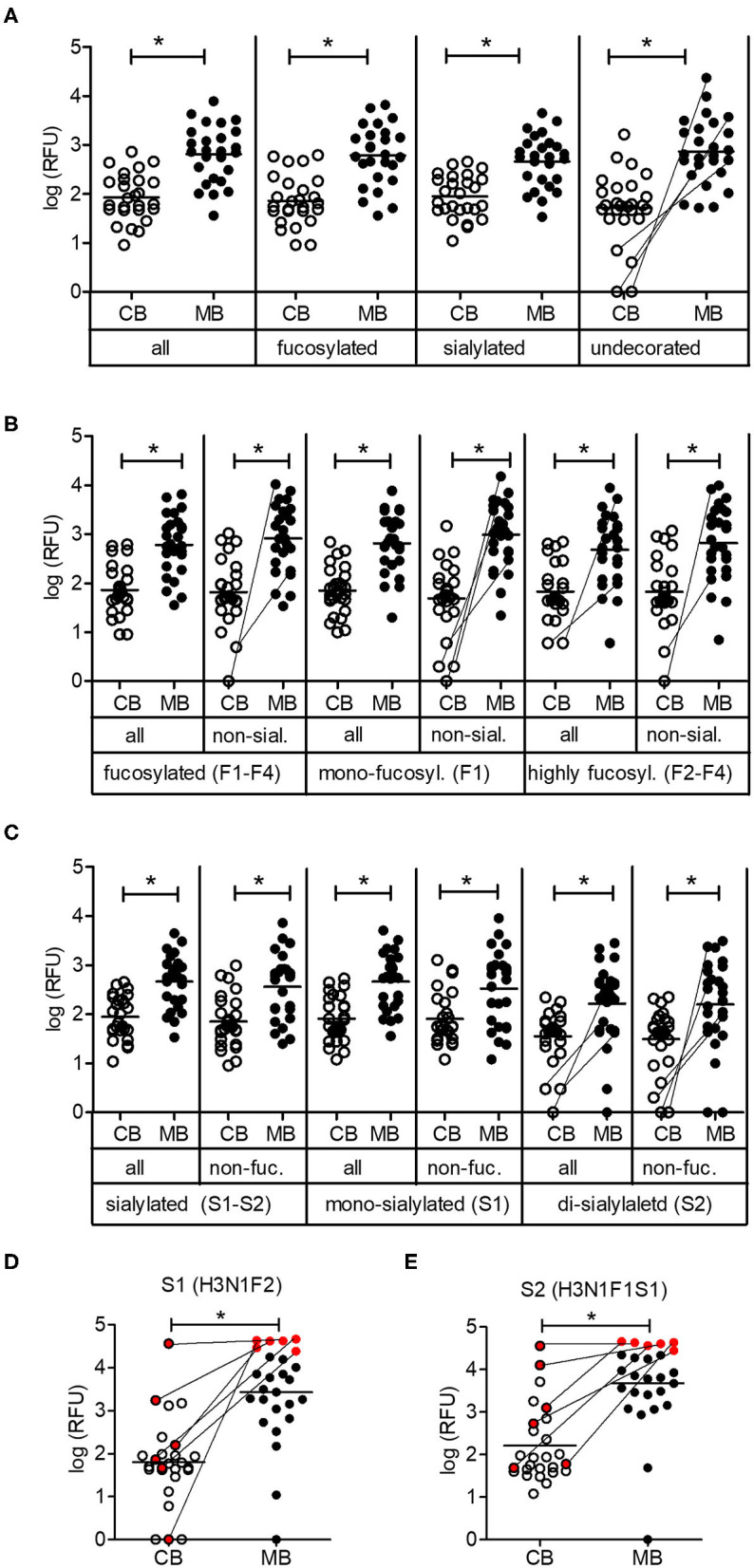
Low maternal transfer of carbohydrate-specific IgG. **(A)** Mean IgG reactivities to subgroups of glycans on the array shown as log (RFU) for cord blood (CB) and maternal blood (MB) samples. **(B)** Analysis of subgroups of fucosylated glycans (mono-fucosylated, F1; di- to tetra-fucosylated, F2–F4) **(C)** Analysis of subgroups of sialylated glycans (mono-sialylated, S1; di-sialylated, S2). **(D,E)** IgG reactivities of CB and MB samples to glycan structures S1 and S2. A subgroup of samples with comparable maternal IgG reactivity is marked in red. The composition of glycans is indicated by the number of different monosaccharides (hexose, H; HexNAc, N; fucose, F; sialic acid, S). All panels comprise 26 matching cord blood samples and maternal blood samples. Connecting lines between CB and MB dots highlight matching samples with low IgG reactivity in CB. Results with *p*-value < 0.05 are marked with asterisks.

The partition of oligosaccharides into fucosylated, sialylated and undecorated structures did not reveal any major differences in reactivity between cord blood and maternal blood IgG. When focusing on non-fucosylated and non-sialylated oligosaccharides, some cord blood samples contained only marginal levels of IgG recognizing such undecorated oligosaccharides, whereas the corresponding maternal blood samples displayed strong reactivity toward this group of oligosaccharides (Figure 3A). Similar differences were visible for the abundance of IgG reactive toward individual groups of fucosylated oligosaccharides, where IgG reactivity was absent or extremely low in cord blood samples despite a high IgG reactivity in the matched maternal samples (Figure 3B). The same trend was observed for di-sialylated oligosaccharides, regardless of the fucosylation status (Figure 3C). Focusing on single glycan structures, we selected the glycans with the highest average IgG reactivity in the cord blood group. Two glycans displayed a mean RFU higher than 1,000 for the cord blood group and additional strong IgG reactivity for 24 out of 26 maternal blood samples (Figures 3D,E). Interestingly, strong maternal IgG reactivity did not always lead to high levels of IgG binding in matched cord blood, as illustrated by a subgroup of samples (Figures 3D,E). For six maternal samples with a comparable and strong IgG reactivity to structure S1, only one matched cord blood sample displayed the same strength in IgG binding to the same glycan. Compared with the matched maternal samples, the other five cord blood samples resulted in weaker IgG binding, still leading to strong recognition of structure S1 in one case and weak responses for three samples. Interestingly, despite the high levels of IgG reactivity observed in the matched maternal blood, one cord blood sample did not show any IgG binding to the same glycan (Figure 3D). A similar pattern was observed for the second glycan structure S2 (Figure 3E). The discrepancy in the IgG reactivity between cord blood and matching maternal blood samples pointed to a selective transfer of maternal carbohydrate-specific IgG to the fetal circulation.

**Figure 4:**
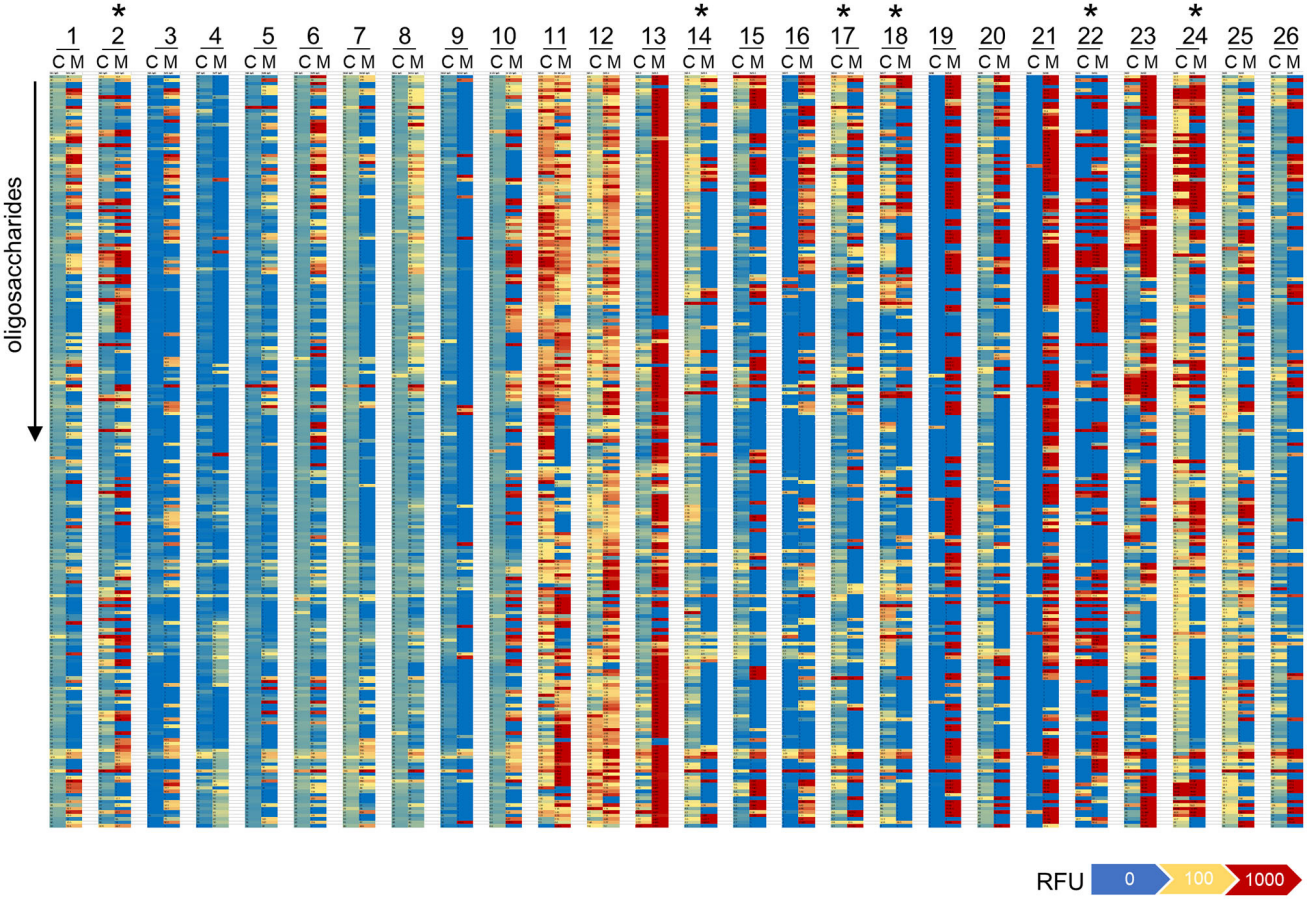
Repertoires of carbohydrate-specific IgG only partially overlap in matched maternal blood and cord blood. Heatmaps representing the profile of carbohydrate-specific IgG in 26 matched pairs of cord blood (C) and maternal blood (M). Pairs with visible overlaps in the IgG repertoires are indicated by an asterisk.

The direct comparison of IgG reactivity profiles toward oligosaccharide antigens in the 26 matched pairs of cord blood and maternal blood samples confirmed the general lack of correlation in antigen specificity. Only six pairs displayed visible overlaps in IgG binding patterns (Figure 4). Correlation analysis of normalized IgG reactivities demonstrated that carbohydrate-specific IgG profiles of maternal and cord blood strongly correlated (*r* > 0.67) only for two of 26 pairs (CB2/MB2, CB22/MB22), whereas three pairs showed a moderate correlation (*r* = 0.36–0.67) and 21 pairs did not or only weakly correlate (39) (Table 1).

**Table 1:** Correlation of IgG reactivities to glycans in matched cord blood and maternal blood samples.

**Sample**	***r*^**a**^**	***P*-value**
CB1/MB1	0.2986	6.91E-06
CB2/MB2	0.8110	2.00E-52
CB3/MB3	0.1861	0.0057
CB4/MB4	0.2430	0.0003
CB5/MB5	0.0962	0.1559
CB6/MB6	0.0207	0.7609
CB7/MB7	0.4480	3.29E-12
CB8/MB8	0.1576	0.0196
CB9/MB9	0.1640	0.0151
CM10/MB10	0.0401	0.5551
CB11/MB11	−0.1052	0.1205
CB12/MB12	0.0064	0.9248
CB13/MB13	0.1504	0.0261
CB14/MB14	0.4586	8.65E-13
CB15/MB15	0.3538	7.43E-08
CB16/MB16	0.1064	0.1165
CB17/MB17	0.3078	3.45E-06
CB18/MB18	0.3057	4.05E-06
CB19/MB19	0.0117	0.8629
CB20/MB20	0.2469	0.0002
CB21/MB21	0.0518	0.4454
CB22/MB22	0.7167	8.09E-36
CB23/MB23	0.2510	0.0002
CB24/MB24	0.6201	1.16E-24
CB25/MB25	0.0730	0.2824
CB26/MB26	0.2993	6.55E-06

a *r, Pearson correlation coefficient*.

Limited transfer of carbohydrate-specific antibodies may be explained by differences in IgG subclasses specifically recognizing carbohydrate antigens. IgG2 is the major subclass of IgG induced to carbohydrate antigens (40, 41), even though other subclasses are involved in anti-carbohydrate responses (42). As the placental transport of IgG2 is less efficient compared with the transport of other IgG subclasses (43), limited transfer of carbohydrate-specific antibodies may be linked to limited transfer of the IgG2 subclass. Testing IgG2 concentrations in cord blood and maternal blood, we determined the IgG2 transfer rate for the 26 pairs by calculating the proportion of IgG2 in cord blood to IgG2 in matched maternal blood (44). The mean IgG2 transfer rate of 95.5 ± 51.5% was lower than total IgG transfer rate of 120.5 ± 52.5% (Supplementary Figure 2).

### Efficient Maternal Transfer of IgG Directed to Commensal Bacterial Antigens

**Figure 5:**
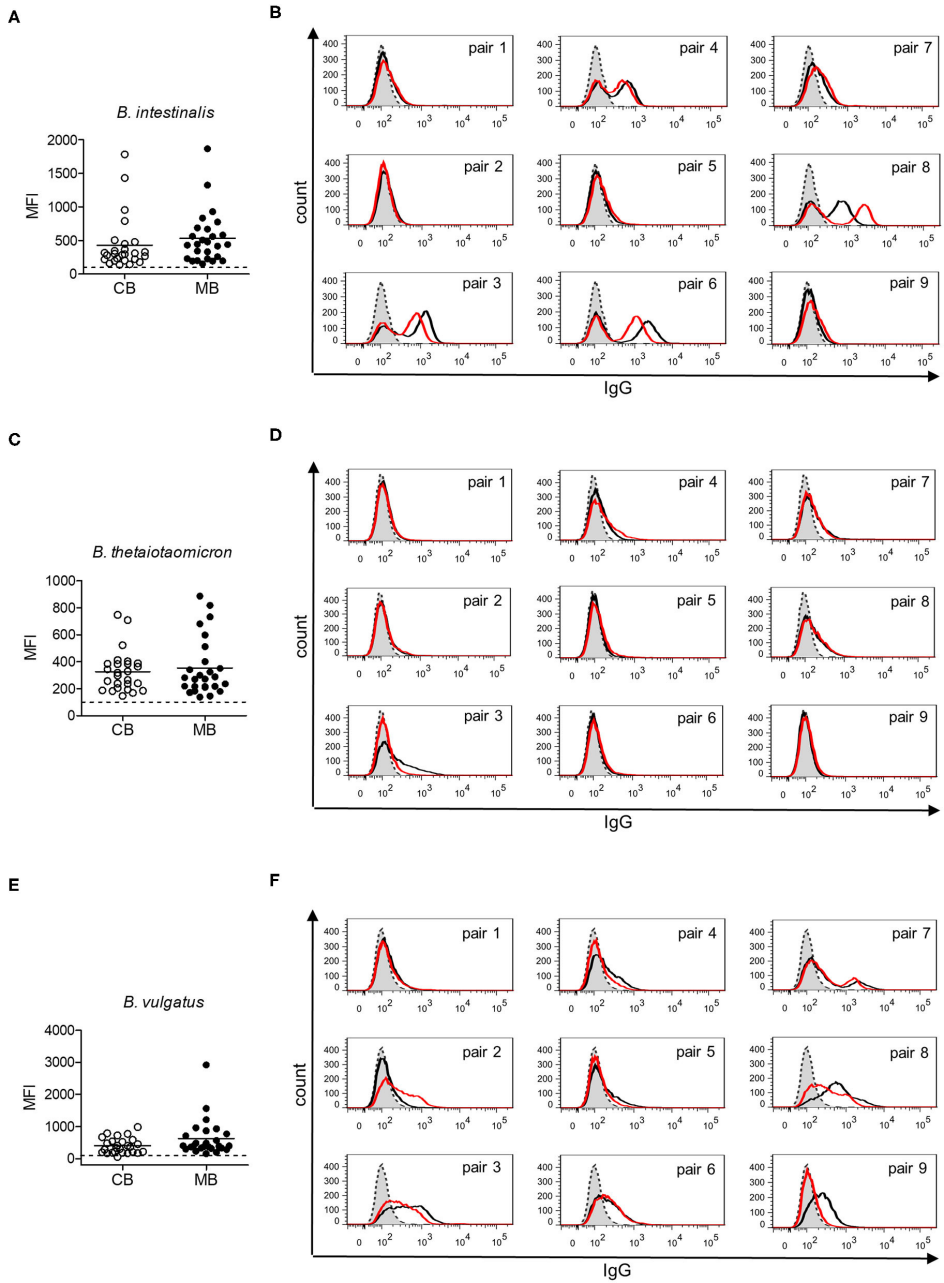
IgG response to commensal intestinal bacteria is comparable in matched cord blood and maternal blood samples. **(A)** IgG reactivity to *B. intestinalis* for cord blood (CB) and maternal blood (MB) samples (*n* = 26). **(B)** IgG reactivity to *B. intestinalis* for matched pairs of cord blood (red) and maternal blood (black). Untreated bacteria are displayed in gray (dashed line). Histograms are related to data presented in **(A)**. **(C)** IgG reactivity to *B. thetaiotaomicron* for cord blood (CB) and maternal blood (MB) samples (*n* = 26). **(D)** IgG reactivity to *B. thetaiotaomicron* for matched pairs of cord blood (red) and maternal blood (black). Untreated bacteria are displayed in gray (dashed line). Histograms are related to data presented in **(C)**. **(E)** IgG reactivity to *B. vulgatus* for cord blood (CB) and maternal blood (MB) samples (*n* = 26). **(F)** IgG reactivity to *B. vulgatus* for matched pairs of cord blood (red) and maternal blood (black). Untreated bacteria are displayed in gray (dashed line). Histograms are related to data presented in **(E)**.

**Figure 6:**
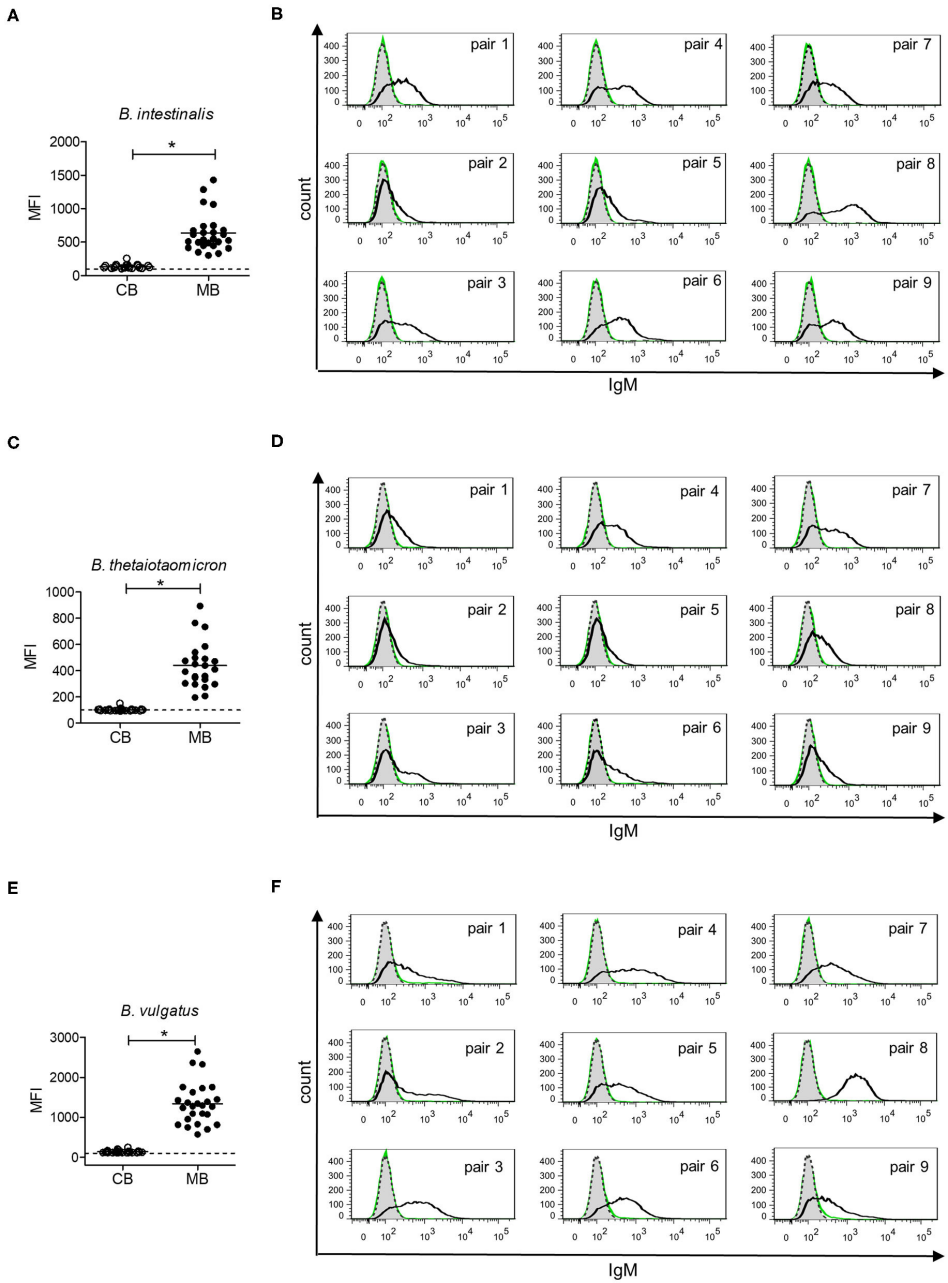
IgM response to commensal intestinal bacteria is absent in cord blood. **(A)** IgM reactivity to *B. intestinalis* for cord blood (CB) and maternal blood (MB) samples (*n* = 25). **(B)** IgM reactivity to *B. intestinalis* for matched pairs of cord blood (green) and maternal blood (black). Untreated bacteria are displayed in gray (dashed line). Histograms are related to data presented in **(A)**. **(C)** IgM reactivity to *B. thetaiotaomicron* for cord blood (CB) and maternal blood (MB) samples (*n* = 23). **(D)** IgM reactivity to *B. thetaiotaomicron* for matched pairs of cord blood (green) and maternal blood (black). Untreated bacteria are displayed in gray (dashed line). Histograms are related to data presented in **(C)**. **(E)** IgM reactivity to *B. vulgatus* for cord blood (CB) and maternal blood (MB) samples (*n* = 26). **(F)** IgM reactivity to *B. vulgatus* for matched pairs of cord blood (green) and maternal blood (black). Untreated bacteria are displayed in gray (dashed line). Histograms are related to data presented in **(E)**. Results with *p*-value < 0.05 are marked with asterisks.

**Figure 7:**
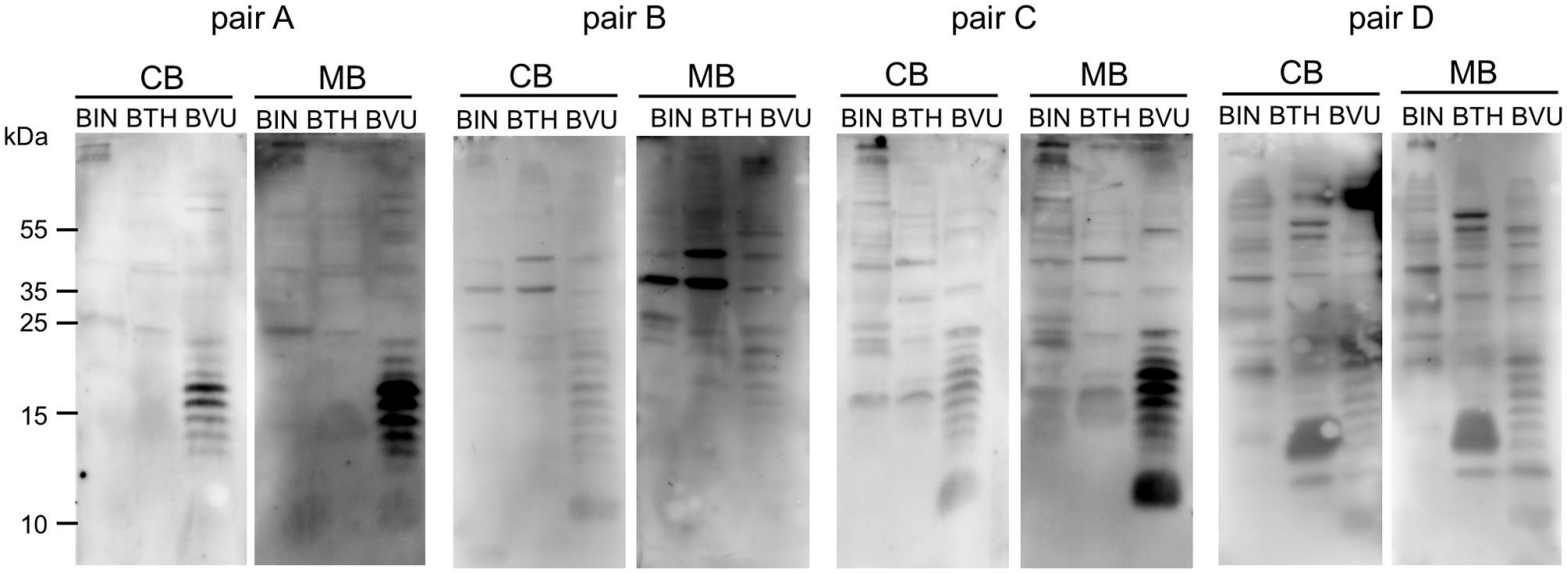
IgG of cord blood and matched maternal blood target identical antigens on commensal bacteria. IgG binding to components of bacterial cell lysates of *B. intestinalis* (BIN), *B. thetaiotaomicron* (BTH), and *B. vulgatus* (BVU) for matched pairs of cord blood and maternal blood, using a serum dilution of 1:500.

Gut bacteria play an important role in the emergence of carbohydrate-specific antibodies (3, 45). Maternal IgG significantly contribute to the protection of the neonate from bacterial infection through placental transfer to the fetal circulation (15–18). To determine whether the selective placental transfer of IgG detected for carbohydrate antigens also affected the recognition of commensal bacteria by cord blood IgG, we compared the reactivity of matched cord blood and maternal blood samples to representative species of commensal *Bacteroides* bacteria. The phylum of Bacteroidetes is one of the major phyla of the human gut microbiota including a variety of species, where the *Bacteroides* are known for their high diversity in surface glycoconjugates (11, 46). All matched cord blood and maternal samples tested showed similar global IgG reactivities to *B. intestinalis, B. thetaiotaomicron*, and *B. vulgatus* (Figures 5A–E), where mean IgG reactivities for maternal and cord blood were on the same level (Figures 5A,C,E). Also the patterns of reactivity between matched pairs of cord blood and maternal blood samples overlapped as assessed by flow cytometry histograms (Figures 5B,D,F). The similarity in antibody reactivity toward the *Bacteroides* species clearly showed that maternal IgG targeting bacterial antigens were efficiently transferred to the fetal circulation. By contrast, IgM reactivity to these three *Bacteroides* species was absent in cord blood samples, whereas elevated reactivities were measured in maternal blood samples (Figures 6A,C,E). The comparison of individual matching cord blood and maternal blood pairs clearly underlined the lack of IgM transfer from the maternal circulation to the fetus (Figures 6B,D,F). The absence of IgM specific to the *Bacteroides* species tested in cord blood also indicated that the fetal immune system was not primed to these commensals prior to delivery, thus supporting the idea that prenatal exposure to gut microbes is insignificant and insufficient to stimulate the production of specific antibodies. To further document the similarity in antigen recognition between matched neonatal and maternal blood derived IgG, the reactivity of selected matched samples toward *Bacteroides* cell lysates was investigated using Western blotting. This analysis confirmed the strong inter-individual variability in antigen recognition between maternal blood samples, as shown by the different patterns of bands recognized by individual samples (Figure 7). The analysis also demonstrated the similarity in antigen recognition between matched cord blood and maternal blood samples, thereby underlining the placental transfer of maternal IgG to commensal bacteria into the fetal circulation.

## Discussion

In this study, we showed that maternal carbohydrate-specific IgG are only partially transferred to the fetus, whereas IgG directed to bacterial antigens in general are efficiently passed from the mother to the fetus prior delivery. We also showed that carbohydrate-specific IgM are lacking at birth, reflecting the absence of maternal transfer for this class of immunoglobulins and the lack of prenatal IgM production. In line with previous studies, IgG concentrations of cord blood correlated with gestational age and reached maternal levels in full-term neonates (12, 23, 43). Interestingly, high levels of maternal IgG led to lower levels of cord blood IgG, suggesting that FcRn receptor-mediated placental transfer can be saturated (43).

Carbohydrate-specific IgG reactivity in cord blood was generally low in comparison with maternal blood, which presented diverse and specific repertoires of carbohydrate-specific IgG, as reported previously (47). Reactivity to carbohydrate antigens only correlated for few pairs of matched cord blood and maternal blood samples. Most cord blood samples tested showed a low and diffuse glycan reactivity, indicative of a limited pool of maternal IgG transferred to the fetal circulation. Limited transfer of carbohydrate-specific antibodies may be partially explained by decreased affinity of the FcRn receptor for IgG2 (20), which have been shown to encompass a large pool of carbohydrate-specific IgG (40, 41). As the mean transfer rate of IgG2 in our study was close to 100%, limited transfer of IgG2 alone cannot explain the strong differences in IgG response to carbohydrates in maternal and cord blood. Additional factors, such as varying Fc region glycosylation and consequent alteration of the binding strength to different Fc receptors, may be responsible for limited transfer of anti-carbohydrate antibodies (44). Of the Fc receptors expressed in placental tissue only the role of FcRn is clear. Other noncanonical Fc receptors, including FcγRI, FcγRII, and FcγRIII, are widely unexplored and may contribute to IgG transport (48). A recent study pointed to preferential transplacental transfer of antibodies with digalactosylated Fc glycans, binding selectively to FcRn and FcγRIIIa. These antibodies activate NK cells, which in contrast to other immune cells, are already fully competent in cord blood, leading to the transfer of antibodies with the best functional potential in the neonatal immune system (49). In addition, placental transfer of IgG may not be the only mechanism of maternal IgG transfer, as IgG from the amniotic fluid could be taken up by the gastrointestinal route (50). Despite the occurrence of large pools of diverse carbohydrate-specific antibodies in the blood serum of adults and the immunogenicity of bacterial polysaccharides, the general low IgG reactivity to glycans in cord blood indicates a minor role of carbohydrate-specific antibodies in the protection of neonates from pathogens.

To date, studies on the maternal transfer of anti-bacterial antibodies to cord blood have focused on pathogenic bacteria in the context of vaccination (15–18) and infections with *Neisseria gonorrhoeae, Salmonella typhosa* and different strains of *Escherichia coli* (21, 51). Our results demonstrate that antibodies to commensal bacteria of the genus *Bacteroides* are also transferred efficiently from the maternal to the fetal circulation. We did not only detect broad IgG responses to different strains of *Bacteroides* in cord blood, but also showed the consistent overlap of bacterial antigens recognized by IgG of cord blood and matched maternal blood. Besides a potential differential Fc glycosylation of different antibodies, the difference in transport efficiency compared with carbohydrate-specific IgG may indicate an involvement of different bacterial antigens (19), which include proteins next to glycans. Slight differences in the IgG binding of matched cord and maternal samples as well as differences in the overall reactivity to the different *Bacteroides* species may account for different bacterial antigens targeted. Accordingly, Western blot data pointed to the presence of protein- and lipopolysaccharide-specific IgG.

The transfer of antibodies recognizing pathogenic bacteria significantly contributes to the protection of newborns from infections. The rationale for the transfer of antibodies to commensal bacteria is however less evident. A possible effect is likely linked to the protection from translocated (52) or potentially pathogenic bacteria (53, 54) from the intestinal milieu to the blood circulation. Furthermore, antibodies originally directed to commensal bacteria, can cross-react and recognize additional bacterial species, including pathogenic strains, through antigen mimicry that is widespread across glycoconjugate antigens (55). Secretory IgA provided through the breast milk also significantly contribute to shaping the gut microbiota (56). Given that FcRn is expressed in human intestinal epithelial cells and has been suggested to transport serum IgG to the intestinal lumen (57–60), serum IgG of maternal origin are also likely able to influence the expansion of the gut microbiota. Prenatally transferred maternal IgG protects the newborn only during the first months of life, as IgG levels start to decrease usually between 2 and 6 months of age (61, 62). Accordingly, the production of highly specific antibodies by the infant immune system only starts postnatally (63). Despite the lack of significant prenatal immunoglobulin production, probably due to a high fetal immune tolerance (64), low and gestational age-dependent IgM concentrations were detected in cord blood in our study and in previous studies (23, 24). Analyzing IgM reactivity to oligosaccharide antigens and commensal bacteria, however, we did not observe any IgM reactivity in cord blood samples. The lack of reactivity toward oligosaccharides support the notion that the fetal immune system is not significantly exposed to microbes prenatally, which could lead to the emergence of primary IgM recognizing carbohydrate antigens. Our study confirms that carbohydrate-specific and commensal-specific IgM only develop postnatally. Analysis of blood and stool samples from infants at different ages would allow to trace the postnatal emergence of carbohydrate-specific IgM and to correlate the occurrence of such antibodies with the process of gut microbial colonization. The further study of the induction of carbohydrate-specific antibodies and the time point of their occurrence in human blood is important, as such antibodies play critical roles in the protection against infections (6, 7) and in numerous diseases, thereby providing a pool of potential biomarkers for diseases and medical applications (65–67).

## Materials and Methods

### Materials

Goat anti-human IgG A488 and goat anti-human IgM A647 were purchased from Jackson ImmunoResearch Laboratories (West Grove, PA). Anti-human IgG DyLight488 and anti-human IgM DyLight488 were obtained from Abcam (Cambridge, MA). Anti-human IgG HRP was from Promega (Fitchburg, WI). *Bacteroides vulgatus* (DSM 1447), *Bacteroides intestinalis* (DSM 17393), and *Bacteroides thetaiotaomicron* (DSM 2079) were obtained from the German Collection of Microorganisms and Cell Cultures (DSMZ, Braunschweig, Germany).

### Patients and Serum Samples

The study was approved by the cantonal Ethics Committee of Zurich (KEK Nr. 2019-00150). All experiments were performed in accordance with relevant guidelines and regulations. Informed consent was obtained from all participants and/or their legal guardians. Leftover maternal and cord blood samples were obtained from the University hospital Zurich, Switzerland. The study population comprised 30 full-term and 12 pre-term neonates. Out of these 42 samples, matched maternal blood was available for 26 full-term and 5 pre-term cord blood samples. Blood was centrifuged at 3,000 x g for 15 min at 4°C and sera were frozen at −20°C. IgG and IgM concentrations were determined by ELISA [IgG human ELISA Kit from Abnova (KA3817), IgM human ELISA Kit from Abnova (KA1855), Taipeh, China] according to the manufacturer's protocols. IgG transfer was calculated as proportion of the IgG concentration in cord blood to the IgG concentration in maternal blood ([IgG_cord_/IgG_maternal_]^*^100), according to (44).

### Glycan Arrays

The human milk shotgun glycan microarray (version 223) has been previously described (37) and was provided by the National Center for Functional Glycomics (NCFG), BIDMC, Harvard University. The shotgun glycan microarray slides were stored at −20°C, and before use, the arrays were allowed to come to room temperature in a desiccator. Individual slides were loaded with 8-well format ProPlate Chambers (Grace Bio-Labs, OR, USA) and hydrated with TSM-T [20 mM Tris-HCl pH 7.4, 150 mM NaCl, 2 mM CaCl_2_, 2 mM MgCl_2_ (TSM) with 0.05% Tween]. To test antibody reactivity of individual serum samples, arrays were incubated with serum diluted to 500 μg/ml IgG in binding buffer (TSM-T with 1% BSA) for 1 h at room temperature. After incubation, slides were washed four times with TSM-T and for times with TSM. For detection of IgG, slides were incubated for 1 h at room temperature with 5 μg/ml Alexa488-labeled goat anti-human IgG in binding buffer. After washing as described above, the slides were incubated for 1 h at room temperature with 5 μg/ml Alexa647 goat anti-human IgM and washed as described with a final wash of four times with deionized water. After spin-drying, arrays were scanned at 647 nm and 488 nm using Innoscan 1100 AL (Innopsys, Carbonne, France). A mask representing the layout of the array was fit to each image using MAPIX analysis software version 8.5.0 (Innopsys, Carbonne, France) and the mean fluorescence intensity of each spot was calculated. The background signal was subtracted for each spot, the mean of four replicates was calculated and negative values were set to 1. For further analysis log (RFU) was used. Microarray production and analyses were performed according to the MIRAGE guidelines (68) (Supplementary Material).

### Bacterial Cultures

*Bacteroides* species were cultivated anaerobically in rubber-sealed hungate tubes at 37°C in Peptone Yeast Glucose medium (DSMZ, medium no. 104 with 1.25 mg/ml glucose). At OD_600_ of 1.0 cells were harvested in aliquots of 0.5 ml. Cell pellets were received by centrifugation at 13,000 x g for 1 min, and phosphate-buffered saline (PBS) washed pellets were either stored at −20°C for cell lysates or fixed with 2% paraformaldehyde in PBS for 15 min.

### Flow Cytometry

Fixed bacteria were treated with diluted serum (1:100 for IgG, 1:10 for IgM) in PBS for 1 h at room temperature, washed twice using PBS, stained with anti-human IgG DyLight488 or anti-human IgM DyLight488 for 1 h at room temperature and washed twice with PBS. The fluorescence signal was recorded by a FACSCanto II Flow cytometer (BD Biosciences, Franklin Lakes, NJ) and analysis was performed with the FlowJo software (BD Bioscience).

### Western Blots

Bacterial cell lysates were prepared from bacterial pellets, resulting from 0.5 ml culture at OD_600_ of 1.0, resuspended in 200 μl SDS buffer (2% β-mercaptoethanol, 2% SDS, 10% glycerol in 50 mM Tris-HCl, bromophenol blue, pH 6.8), boiled at 99°C for 15 min. Fifteen microliter bacterial cell lysates were run on 14% acrylamide gels at 80 V for 2 h and transferred onto PVDF at 250 mA for 1 h. Membranes were blocked overnight at 4°C with 5% BSA in PBS-T (PBS with 0.1% Tween20). Membranes were incubated with diluted serum in PBS-T (1:500) for 2 h at room temperature, washed four times for 5 min using PBS-T, incubated with anti-human IgG-HRP (1:5,000) in PBS-T for 1 h at room temperature, washed four times for 5 min with PBS-T and detected using SuperSignal® West Pico chemiluminescence substrate (Thermo Fisher Scientific, Waltham, MA) and a Fujifilm LAS-4000 luminescence image analyzer (GE Healthcare, Chicago, IL). Matched cord and maternal blood samples were used on the same membrane, which was cut after transfer, and detection of membrane pieces incubated with matched samples was performed simultaneously.

### Statistical Analysis

Statistical analysis was performed using GraphPad Prism (GraphPad software, San Diego, CA). For the comparison of two groups of paired samples (cord blood vs. maternal blood, IgG total vs. IgG2), either a paired student's *t*-test for Gauss-distributed or a Wilcoxon matched pairs signed rank test for non-Gauss distributed samples was used. For unpaired groups (pre-term vs. full-term), an unpaired *t*-test with Welch's correction was used. Results with *p*-value ≤ 0.05 were considered significantly different and marked with asterisks. For quantile normalization and correlation analysis a software tool run on R (version V3.6.1) using the “limma” package (69) with the functions cor.test() and normalizeQuantiles() was used.

## Data Availability Statement

The raw data supporting the conclusions of this article will be made available by the authors, without undue reservation.

## Ethics Statement

The studies involving human participants were reviewed and approved by the cantonal Ethics Committee of Zurich (KEK Nr. 2019-00150). All experiments were performed in accordance with relevant guidelines and regulations. Informed consent was obtained from all participants and/or their legal guardians. Written informed consent to participate in this study was provided by the participants' legal guardian/next of kin.

## Author Contributions

TH designed the study and secured the funding. TR and DB submitted the ethical approval and organized the blood samples. KK performed and analyzed the experiments. YL performed the array experiments. KK and TH wrote the manuscript. TR, DB, DS, and YL revised the manuscript. All authors contributed to the article and approved the submitted version.

## Conflict of Interest

The authors declare that the research was conducted in the absence of any commercial or financial relationships that could be construed as a potential conflict of interest.
