# Supplementary material for: Limited Neonatal Carbohydrate-Specific Antibody Repertoire Consecutive to Partial Prenatal Transfer of Maternal Antibodies
Source: Front Immunol. 2020 Oct 14;11:573629. doi: 10.3389/fimmu.2020.573629 (PMC7591393; doi:10.3389/fimmu.2020.573629)
Supplement: Supplementary file 1 [file Table_1.DOCX]

Supplementary Material

## Supplementary Tables

| **Table S1.** IgG and IgM concentrations of cord blood samples and matched maternal blood. | | | | | |
| --- | --- | --- | --- | --- | --- |
|  |  | cord blood | | maternal blood | |
| sample | gestational age [weeks] | IgG  [mg/mL] | IgM  [mg/mL] | IgG  [mg/mL] | IgM  [mg/mL] |
| CB1/MB1 | 39 3/7 | 9.92 | 0.56 | 9.44 | 1.12 |
| CB2/MB2 | 37 5/7 | 5.77 | 0.39 | 28.15 | 2.15 |
| CB3/MB3 | 40 6/7 | 16.22 | 0.55 | 8.55 | 0.89 |
| CB4/MB4 | 38 1/7 | 8.63 | 0.54 | 16.31 | 1.42 |
| CB5/MB5 | 40 2/7 | 20.64 | 0.68 | 12.30 | 1.31 |
| CB6/MB6 | 40 | 20.43 | 0.91 | 11.89 | 1.09 |
| CB7/MB7 | 40 | 15.98 | 0.61 | 23.88 | 1.20 |
| CB8/MB8 | 39 | 18.88 | 0.63 | 16.14 | 1.17 |
| CB9/MB9 | 38 3/7 | 14.11 | 0.54 | 15.49 | 1.32 |
| CB10/MB10 | 40 1/7 | 15.96 | 0.80 | 16.77 | 1.43 |
| CB11/MB11 | 38 6/7 | 7.81 | 0.53 | 14.67 | 0.84 |
| CB12/MB12 | 39 3/7 | 17.91 | 0.70 | 9.37 | 0.84 |
| CB13/MB13 | 40 4/7 | 16.65 | 0.69 | 12.36 | 0.91 |
| CB14/MB14 | 40 | 18.84 | 0.76 | 12.51 | 0.76 |
| CB15/MB15 | 38 3/7 | 10.34 | 0.66 | 12.40 | 0.70 |
| CB16/MB16 | 41 3/7 | 14.95 | 0.45 | 10.90 | 0.65 |
| CB17/MB17 | 38 6/7 | 6.71 | 0.54 | 12.35 | 0.96 |
| CB18/MB18 | 40 4/7 | 17.74 | 0.71 | 19.14 | 0.82 |
| CB19/MB19 | 39 1/7 | 10.52 | 0.55 | 11.07 | 0.96 |
| CB20/MB20 | 38 2/7 | 25.00 | 0.53 | 12.68 | 1.15 |
| CB21/MB21 | 41 | 13.08 | 0.53 | 20.82 | 1.11 |
| CB22/MB21 | 39 3/7 | 15.16 | 0.60 | 10.72 | 0.67 |
| CB23/MB23 | 38 1/7 | 10.50 | 0.54 | 5.57 | 0.72 |
| CB24/MB24 | 38 | 16.55 | 0.59 | 11.02 | 0.84 |
| CB25/MB25 | 38 4/7 | 15.97 | 0.72 | 13.29 | 1.03 |
| CB26/MB26 | 37 1/7 | 14.88 | 0.55 | 7.32 | 0.97 |
| CB27 | 40 3/7 | 5.95 | 0.51 | n.a. | n.a. |
| CB28 | 38 | 13.54 | 0.76 | n.a. | n.a. |
| CB29 | 40 1/7 | 23.98 | 0.96 | n.a. | n.a. |
| CB30 | 37 1/7 | 9.98 | 0.69 | n.a. | n.a. |
| CB31/MB31 | 26 2/7 | 2.65 | 0.19 | 12.42 | 0.77 |
| CB32/MB32 | 35 2/7 | 15.40 | 0.59 | 13.91 | 0.88 |
| CB33/MB33 | 33 5/7 | 4.27 | 0.34 | 15.17 | 1.15 |
| CB34/MB34 | 34 1/7 | 2.56 | 0.14 | 7.32 | 0.87 |
| CB35/MB35 | 35 4/7 | 8.12 | 0.42 | 13.03 | 0.78 |
| CB36 | 33 3/7 | 8.07 | 0.63 | n.a. | n.a. |
| CB37 | 28 5/7 | 3.69 | 0.30 | n.a. | n.a. |
| CB38 | 26 1/7 | 3.05 | 0.33 | n.a. | n.a. |
| CB39 | 34 1/7 | 11.11 | 0.36 | n.a. | n.a. |
| CB40 | 29 6/7 | 5.59 | 0.33 | n.a. | n.a. |
| CB41 | 31 4/7 | 5.25 | 0.41 | n.a. | n.a. |
| CB42 | 33 5/7 | 11.68 | 0.72 | n.a. | n.a. |

| **Supplementary Table S2.** Correlation of IgG and IgM reactivity to glycans in maternal blood samples. | | | |
| --- | --- | --- | --- |
| sample | r | P-value |  |
| MB1 | 0.9673 | 2.52E-131 |  |
| MB2 | 0.9487 | 1.92E-110 |  |
| MB3 | 0.9442 | 1.26E-106 |  |
| MB4 | 0.8453 | 5.28E-61 |  |
| MB5 | 0.8512 | 1.09E-62 |  |
| MB6 | 0.9090 | 2.01E-84 |  |
| MB7 | 0.9078 | 7.69E-84 |  |
| MB8 | 0.8856 | 3.18E-74 |  |
| MB9 | 0.7943 | 7.35E-49 |  |
| MB10 | 0.9752 | 3.76E-144 |  |
| MB11 | 0.9321 | 1.07E-97 |  |
| MB12 | 0.7619 | 8.35E-43 |  |
| Mb13 | 0.9292 | 9.74E-96 |  |
| MB14 | 0.8261 | 5.82E-56 |  |
| MB15 | 0.8154 | 2.07E-53 |  |
| MB16 | 0.7797 | 5.33E-46 |  |
| MB17 | 0.8489 | 5.12E-62 |  |
| MB18 | 0.6755 | 1.54E-30 |  |
| MB19 | 0.9801 | 1.78E-154 |  |
| MB20 | 0.9127 | 2.77E-86 |  |
| MB21 | 0.9239 | 1.78E-92 |  |
| MB22 | 0.9701 | 1.72E-135 |  |
| MB23 | 0.9762 | 5.47E-146 |  |
| MB24 | 0.9134 | 1.14E-86 |  |
| MB25 | 0.9528 | 2.56E-114 |  |
| MB26 | 0.9063 | 4.22E-83 |  |
|  |  |  |  |
| r = Pearson correlation coefficient | | |  |

## Supplementary Figures

##
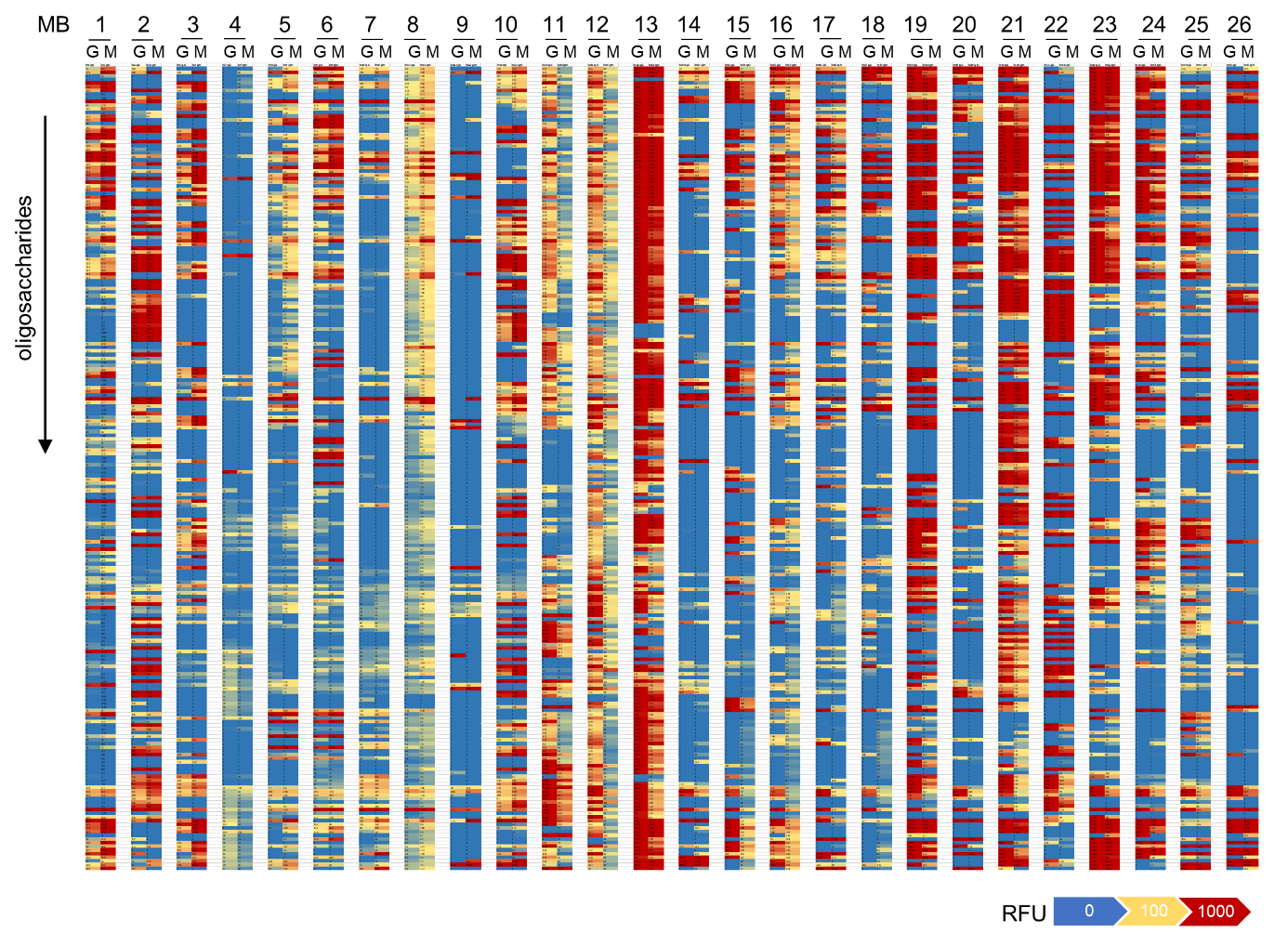


**Supplementary Figure S1:** Broad overlap of the carbohydrate-specific IgG and IgM repertoires in maternal blood samples. Heatmaps representing the profile of carbohydrate-specific IgG (G) and IgM (M) in 26 maternal blood samples (MB1-MB26).

**
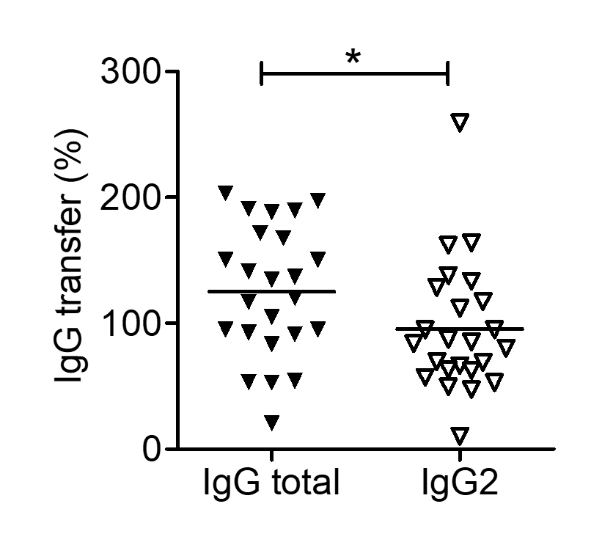
**

**Supplementary Figure S2:** Maternal transfer of IgG2 is lower than transfer of total IgG. IgG2 transfer rate, calculated as proportion of IgG2 in cord blood to IgG2 in matched maternal blood, in comparison with transfer rate of total IgG (n=24).

**ECGC Supplemental glycan microarray document based on MIRAGE Guidelines (doi:10.3762/mirage.3)**

**Array Name from Vendor**: HUMAN MILK GLYCAN 223

**Source**: Glycomics Core/BIDMC/Harvard Medical School

**Array name assigned by ECGC:** HMO223

**Date received**: 7/18/2017

**Slide#:** 25

**Labeled Index**: In-printed Barcode

| **Classification** | |
| --- | --- |
| 1. Sample: Glycan Binding Sample | |
| Description of Sample | human blood serum |
| Sample modifications | diluted to 500 µg/ml IgG |
| Assay protocol | After hydrating with TSM-T, arrays were incubated with serum diluted to 500 µg/ml IgG in binding buffer (TSM-T with 1% BSA) for 1 h at room temperature, washed (4 x TSMT-T, 4 x TSM) and incubated for 1 h at room temperature with 5 µg/ml Alexa488-labeled goat anti-human IgG in binding buffer. After washing as described above, the slides were incubated for 1 h at room temperature with 5 µg/ml Alexa647 goat anti-human IgM and washed as described with a final wash of four times with deionized water and spin-dried. |
| 2. Glycan Library | |
| Glycan description for defined glycans | Defined glycans were purchased from Elicityl.   \| 3'-Fucosyllactose / 3'-FL (GLY060) \| \| --- \| \| Lacto-N-tetraose / LNT (GLY010) \| \| Lacto-N-neotetraose / LNnT (GLY021) \| \| Blood Group H antigen pentaose type I / Lacto-N-fucopentaose I / LNFP I (GLY033-1) \| \| Lewis Y (LeY) pentaose (GLY052) \| \| Lacto-N-fucopentaose III / LNFP III \| \| Lacto-N-neofucopentaose / LNnFP V / LNFP IV (GLY061) \| \| Lacto-N-fucopentaose V / LNFP V (GLY062) \| \| Sialyl-lacto-N-tetraose a / LSTa (GLY081) \| \| Sialyl-lacto-N-tetraose b / LSTb \| \| Sialyl-lacto-N-tetraose a / LSTc \| \| Sialylated tetraose type I / Sialyl Lewisa precursor (GLY080) \| \| Lewis X (LeX) tetraose (GLY050) \| |
| Glycan description for undefined glycans | Human milk glycans were isolated from skimmed milk. |
| Glycan modifications | Glycans were conjugated with AEAB and separated by HPLC. See Yu *et al.* 2012 and Yu *et al.* 2014 for shotgun glycan preparation schema. |
| 3. Printing Surface; e.g., Microarray Slide | |
| Description of surface | Schott type H slides |
| Manufacturer | SCHOTT North America, Inc., Elmsford, NY |
| Custom preparation of surface | No |
| Non-covalent Immobilization | Yes |
| Covalent Immobilization | No |
| 4. Arrayer (Printer) | |
| Description of Arrayer | Scienion sciFLEXARRAYER S11 Microarray Printer |
| Dispensing mechanism | TGL glycans were dispensed at ~333 picoliter per spot to produce ~ 110 micron diameter spots. |
| Glycan deposition | TGL glycans were dispensed at various concentration (see appendix I) in sodium phosphate buffer (100 mM, pH 8.5). |
| Printing conditions | Relative humidity was maintained at 60%. Slides were blocked with 50 mM Ethanolamine in 100 mM Sodium tetraborate buffer (pH 8.5). Printed slides were stored at -20°C before use. |
| 5. Glycan Microarray with “Map” | |
| Array layout | Glycan microarray slides were printed with 8 (1 x 8) subarrays. Each sample was printed in 4 replicates.  Incubation chamber: 8-well format ProPlate Chamber (Grace Bio-Labs, OR, USA). |
| Glycan identification and quality control | Visual inspection of printed spots |
| 6. Detector and Data Processing | |
| Scanning hardware | InnoScan 1100 AL scaner (Innopsys, Carbonne, France) |
| Scanner settings | Scanning resolution: 5 µm/pixel;  Laser channel 635 nm: PMT gain: 35, Power: low;  Laser channel 488 nm: PMT gain: 80, Power: high. |
| Image analysis software | Mapix version 8.2.7. (Innopsys, Carbonne, France) |
| Data processing | The gpr files were processed with an in-house workflow using excel macro. No particular normalization method was used. The background signal was subtracted, the mean of four replicates was calculated (RFU) and negative values were set to 1. |
| 7. Glycan Microarray Data Presentation | |
| Data presentation | Data is presented as mean RFU or log (RFU) of single glycans or subgroups of glycans. Statistical analysis was performed using GraphPadPrism version 5.03 (GraphPad software, San Diego, CA) for one-Way ANOVA and Bonferroni’s multiple comparison test for Gauss distributed samples or Kruskal-Wallis test and Dunn’s post hoc test for non-Gauss distributed samples. Pearson’s correlation analysis was performed using GraphPad Prism version 5.03. To compare the IgG reactivity to single oligosaccharides for patients’ and control groups, a software tool run on R (version V3.6.1) using the “limma” package (Ritchie *et al.* 2015) was used. |
| 8. Interpretation and Conclusion from Microarray Data | |
| Data interpretation | see Results and Discussion |
| Conclusions | see Results and Discussion |

**References:**

Yu, Y, Mishra, S, Song X, Lasanajak Y, Bradley KC, Tappert MM, *et al*. Functional glycomic analysis of human milk glycans reveals the presence of virus receptors and embryonic stem cell biomarkers. Journal of Biological Chemistry. 2012; 287(53):44784-44799. doi:[10.1074/jbc.M112.425819](https://doi.org/10.1074/jbc.M112.425819).

Yu Y, Lasanajak Y, Song X, Hu L, Ramani S, Mickum ML, *et al*. Human milk contains novel glycans that are potential decoy receptors for neonatal rotaviruses. Molecular and Cellular Proteomics. 2014; 13(11):2944-2960. doi: 10.1074/mcp.M114.039875.

Ritchie ME, Phipson B, Wu D, Hu Y, Law CW, Shi W, *et al.* limma powers differential expression analyses for RNA-sequencing and microarray studies. Nucleic acids research. 2015;43(7):e47. doi: 10.1093/nar/gkv007.
